# Smartphone Applications Can Serve as Effective Cognitive Training Tools in Healthy Aging

**DOI:** 10.3389/fnagi.2017.00436

**Published:** 2018-01-04

**Authors:** Blanka Klimova, Martin Valis

**Affiliations:** ^1^Department of Applied Linguistics, Faculty of Informatics and Management, University of Hradec Kralove, Hradec Kralove, Czechia; ^2^Department of Neurology, University Hospital Hradec Kralove, Hradec Kralove, Czechia

**Keywords:** older people, smartphone apps, cognitive training, effectiveness, opinion

## Introduction

Due to the demographic changes, the number of aging population is rapidly rising. This is particularly true for developed countries. For example, in the EU countries in 2016, the share of people aged 65+ years was 19.2%, which was an increase of 0.3% in comparison with the previous years and 2.4% in comparison with 10 years earlier. And it is expected that the share of older people aged 65+ years will rise to 29.1% by 2080 (Eurostat, 2017).

Therefore, governments all over the world try to implement strategies or guidelines for the maintenance or even improvement of quality of life of older people and thus, reduce their and country's economic and social burden. One of the solutions seems to be the use of modern technological devices such as computers and more recently, smartphones. As Pew Research center^1^ states, four in ten seniors now own smartphones, which is twice as many than in 2013. However, there is still an enormous gap in the use of smartphones between the generations. For example, 85% of the French aged 18-34 years own a smartphone compared with 35% of the French who are 65+ years. There is a 50% point difference between these two groups and the same is true for Poland or Germany (Poushter, 2016). Nevertheless, one can observe a gradual growth since in 2015 out of 64% of all American adults, 27% of the Americans aged 65+ owned a smartphone, which was an 8-point increase in comparison with the year of 2014 (Smith, 2015). In Europe, the leading countries among the senior smartphone users are the Scandinavian countries, especially Norway and Sweden. As far as the gender is concerned, senior males tend to use smartphones more frequently than their female counterparts (Berengner et al., 2017).

As it has been already mentioned, the main focus in the aging process concentrates on the maintenance and enhancement of elderly people's quality of life, specifically their cognitive, mental, and physical health (cf. Lu et al., 2017). Particularly the concept of cognitive reserve, which can be defined as the resilience to neuropathological damage of the brain (Stern, 2013), plays an important role in the aging process. It has been argued that this brain reserve is the result of experience-based neural changes which are caused by a physically and mentally stimulating lifestyle, as well as by higher left frontal cortex-connectivity (Freret et al., 2015; Franzmeier et al., 2017a,b; Gelfo et al., 2017). Research studies (e.g., McClearn et al., 1997) reveal that 60% of general cognitive ability is of genetic origin, but there are some non-pharmacological activities such as cognitive training, which may be effective in its enhancement as well (Fox, 2017). Antoniou et al. (2013) report that some of these non-pharmacological activities, which are aimed at the stimulation of the cognitive functions might delay the cognitive decline. Smartphone applications seem to be a good support tool for cognitive training in aging.

Therefore, the purpose of this opinion article is to discuss the potential of smartphone applications (apps) for the enhancement of cognitive functions of older people.

## Recent findings on the use of smartphone apps for cognitive training in healthy aging

Although there are now just a few randomized controlled studies on the use of smartphone apps for the cognitive training of the elderly (Shin et al., 2017), if compared with the smartphone app focused on physical training, the findings indicate that smartphone apps have a promising potential for the enhancement of cognitive competences of older people, specifically for the improvement of their working memory and reasoning skills (Klimova, 2016). In addition, the findings imply that cognitive training via smartphone apps is feasible. For example, Korean study by Oh et al. (2017) and Shin et al. (2017) carried out with 53 individuals aged between 50 and 68 years assessed the effects of smartphone-base cognitive training app aimed at memory. The results of this study showed that the total working memory (WM) quotient considerably had increased [*t*_(17)_ D 6.27, *p* < 0.001], as well as auditory-verbal WM score [*t*_(17)_ D 4.45, *p* < 0.001] after applying the smartphone app for 15-20 min a day for 8 weeks. This was also confirmed by Borella et al. (2013), who argue that there is still room for the elderly to enhance their working memory skills because the results of their study indicate that working memory training programs generate persistent benefits, particularly in the verbal working memory tasks. As Migo et al. (2015) claim, smartphones can serve as an external memory aid.

Furthermore, the Norwegian study by Bless et al. (2014) described the positive effects of smartphone app on self-supervised training of auditory attention in 28 older individuals. The results of a 3-week training indicated an improvement in attention performance, which was accompanied by corresponding change in brain activation. However, transfer of the training to other tasks tapping into similar mechanisms was not observed (cf. Cotton, 2017). Nevertheless, there are a few studies (Štěpánková et al., 2012; Flak et al., 2014), which show that computer-based cognitive training programs might have positive near-transfer effects, both in healthy older individuals and older people with MCI.

Lu et al. (2017) then described in their study the development and evaluation of iPad-based app for cognitive training game used with nine older individuals (61–90 years). Their results revealed that the subjects had been satisfied with the designed cognitive training game app. However, the results also showed that its cognitive structure, interface, interaction, instructions and feedback must be always well-thought of when it comes to its use among the elderly people. As Klimova (2017) points out, training should be provided for these people to avoid social anxiety of using smartphone apps. Moreover, companies developing these apps should be aware of meeting specific needs of their potential older clients whose number will be rapidly rising in the near future (cf. Thorpe et al., 2016).

In addition, there are a few research studies exploring the effect of the smartphone apps focused on physical exercises which may have a positive impact on the improvement of cognitive functions among the elderly people. This is, for example, a study by Shellington et al. (2017), whose HealtheBrain smartphone app delivers square-stepping exercise (SSE). The results generated among 19 adult individuals (aged 59–76 years) after a 3-week training showed that this app might serve as a scalable intervention to promote cognitive health in older adults. Nevertheless, in order to generate some positive effects, cognitive trainings must be performed regularly, intensively, and over a longer time span.

As far as the cognitive functions are concerned, the findings show that at present the smartphone apps are mainly exploited in the assessment of cognitive functions in older people (Shigemori and Okamoto, 2015). For instance, Allard et al. (2014) argue that traditional tools are sometimes unable to detect sensitive declines in cognitive functions due to natural variation at the time of testing. Nevertheless, smartphones can enable the repeated assessments of cognitive functions and might provide more reliable descriptions of early cognitive disorders.

Recent findings also reveal that the smartphone apps may be promising self-management tools for the treatment of depression (cf. Arean et al., 2016; Firth et al., 2017), which is one of the most serious comorbidities in the aging process (Popa-Wagner et al., 2014; Sandu et al., 2015). In fact, depression is the fourth common cause of morbidity and by 2020 it is estimated to seize the first place (Maresova et al., 2017). The reason is not a higher incidence of depression, but rather the consequence of combating somatic diseases which hold primacy in morbidity nowadays such as the case of cardiovascular diseases. The smartphone apps might be beneficial for the reduction of depression symptoms such as anxiety, loneliness, or cognitive behavior. The research shows that mobile apps may be especially useful in the following areas: running online treatment trainings, online interactive psychotherapeutic courses, online cognitive behavioral therapy sessions, online motivational home reminders, online diary writing about problems, or monitoring of the symptoms of depression (cf. Maresova et al., 2017).

## Conclusion

Overall, the findings of this article indicate that the smartphone apps thanks to their independence on time and place may serve as good intervention tools for cognitive training of older individuals. Nevertheless, several factors such as training of the elderly, design of the smartphone, level of difficulty of cognitive training, or security functions, must be carefully considered if they are to be used by these elderly people. In addition, more randomized controlled trials should be done in this field to prove the efficacy of the smartphone apps on cognitive training of older people, as well as to show for which group of the elderly they are most suitable since it seems that the breaking age for the use of smartphones nowadays is 74 years (cf. Simonova et al., 2017).

## Author contributions

BK and MV equally contributed to the drafting, analyses and final version of the whole manuscript. All authors read and approved the final manuscript.

### Conflict of interest statement

The authors declare that the research was conducted in the absence of any commercial or financial relationships that could be construed as a potential conflict of interest.

## Footnotes

^1^Available online at: http://www.pewinternet.org/2017/05/17/technology-use-among-seniors/ (Accessed October 19, 2017).
